# Natural Killer Defective Maturation Is Associated with Adverse Clinical Outcome in Patients with Acute Myeloid Leukemia

**DOI:** 10.3389/fimmu.2017.00573

**Published:** 2017-05-29

**Authors:** Anne-Sophie Chretien, Cyril Fauriat, Florence Orlanducci, Claire Galseran, Jerome Rey, Gaelle Bouvier Borg, Emmanuel Gautherot, Samuel Granjeaud, Jean-François Hamel-Broza, Clemence Demerle, Norbert Ifrah, Catherine Lacombe, Pascale Cornillet-Lefebvre, Jacques Delaunay, Antoine Toubert, Emilie Gregori, Herve Luche, Marie Malissen, Christine Arnoulet, Jacques A. Nunes, Norbert Vey, Daniel Olive

**Affiliations:** ^1^Team Immunity and Cancer, Centre de Recherche en Cancérologie de Marseille (CRCM), Inserm, U1068; CNRS, UMR7258, Institut Paoli-Calmettes; Aix-Marseille University, UM 105, Marseille, France; ^2^Immunomonitoring platform, Institut Paoli-Calmettes, Marseille, France; ^3^Hematology Department, Centre de Recherche en Cancérologie de Marseille (CRCM), Inserm, U1068; CNRS, UMR7258, Institut Paoli-Calmettes; Aix-Marseille University, UM 105, Marseille, France; ^4^Beckman Coulter Immunotech, Marseille, France; ^5^Systems Biology Platform, Centre de Recherche en Cancérologie de Marseille (CRCM), Inserm, U1068; CNRS, UMR7258, Institut Paoli-Calmettes; Aix-Marseille University, UM 105, Marseille, France; ^6^Biostatistics and methodology department, CHU Angers, Angers, France; ^7^Hematology department, CHU Angers, Angers, France; ^8^GOELAMStheque, FILO (French Innovative Leukemia Organization), Cochin Hospital, APHP, Paris, France; ^9^Laboratoire d’Hématologie, Centre Hospitalier Universitaire de Reims, Reims, France; ^10^Service d’Hématologie, Centre Catherine de Sienne, Nantes, France; ^11^INSERM UMRS-1160, Univ Paris Diderot, Sorbonne Paris Cité, Institut Universitaire d’Hématologie, Immunology and Histocompatibility department, Hôpital Saint-Louis, APHP, Paris, France; ^12^Centre d’Immunophénomique – CIPHE (PHENOMIN), Aix Marseille University, UMS3367; Inserm US012; CNRS, UMS3367, Marseille, France; ^13^Centre d’Immunologie de Marseille-Luminy, Aix Marseille Université UM2, Inserm U1104, CNRS UMR7280, F-13288, Marseille, France; ^14^Biopathology Department, Institut Paoli Calmettes, Marseille, France

**Keywords:** acute myeloid leukemia, prognostic biomarkers, natural killer, natural killer maturation, mass cytometry

## Abstract

Accumulating evidence highlights natural killer (NK) cell parameters as potential prognostic factors in cancer patients, which provides a strong rationale for developing therapeutic strategies aiming at restoring NK cell. However, reaching this point warrants better characterization of tumor-induced NK cell alterations. Our group recently reported heterogeneous NK maturation in acute myeloid leukemia (AML) patients. However, the clinical significance of such observations remained to be assessed on a larger cohort of patients. NK maturation based on expression of CD56, CD57, and KIR was assessed by flow cytometry in newly diagnosed AML patients (*N* = 87 patients from GOELAMS-LAM-IR-2006 multicenter trial). Clinical outcome was evaluated with regard to NK maturation profiles. Unsupervised integrated analysis of NK maturation markers confirmed the existence of three distinct groups of patients [hypomaturation (24.1%), intermediate maturation (66.7%), and hypermaturation (9.2%)]. In univariate analysis, significant differences in overall survival (OS) (*P* = 0.0006) and relapse-free survival (RFS) (*P* < 0.0001) were observed among these different groups. Patients with hypomaturation profile had reduced OS, with 3-year OS rates of 12.5 vs 57.1 and 57.4% for patients with intermediate and hypermaturation, respectively. Consistently, patients with hypomaturation profile had reduced RFS, with 3-year RFS rates of 0 vs 52.6 and 73.3% for patients with intermediate and hypermaturation, respectively. In multivariate Cox regression models, NK hypomaturation remained significantly associated with reduced OS and RFS, independent of other factors [hazard ratio (HR) = 4.15, *P* = 0.004 and HR = 8.23, *P* = 0.003, respectively]. NK maturation defects were further explored by mass cytometry and revealed that NK hypomaturation profile is associated with a reduced frequency of memory-like NK cells. In conclusion, besides classical alterations of NK triggering and inhibitory receptors expression in AML, we confirm that the homeostasis of NK maturation can be modified in the context of AML, notably with a deep maturation blockade in almost 10% patients.

## Introduction

Acute myeloid leukemia (AML) is a hematologic malignancy with poor clinical outcome, in particular in patients over 60 years (1). To date, therapeutic options remain limited. Among hallmarks of cancer, escape to the immune system is generally involved in cancer progression (2). Besides, the three “E” theory (3) clearly defines the immune system as a corner stone of tumor progression and aggressiveness, and therefore of the response to treatment and prolonged remission.

Although the mutational burden is lower in AML compared to solid tumors, leukemic blast express leukemia-associated antigens that are recognized by the immune system (4), with natural killer (NK) cells, CD8^+^ T cells and γδ T cells being the most potent immune effectors that mediate leukemic cells recognition and clearance. Clinical evidence for an anti-leukemic immune effect is seen in recipients of allogeneic stem cell transplantation (allo-SCT) (5). However, immune escape occurs through mechanisms partially shared with solid tumors [reviewed in Ref. (4)]. Among others, leukemic blasts express the coinhibitory molecule programed death ligand 1 or suppressive ligands, such as Galectin 9, which precludes T cell activation (6). In addition, T cell evasion pathway have been described in murine models of AML, with CD8^+^ T cell anergy associated with T cell abortive activation with following antigen encounter (7). In humans, aberrant T cell activation patterns have been described in the context of AML, some of which having been associated with clinical outcome and response to stem cell transplantation (8, 9). In addition, immune evasion from T cell in AML is enhanced by accumulation of suppressive immune cells such as regulatory T cells (Tregs) (10, 11) as well as myeloid-derived suppressive cells (MDSCs) (12).

Besides, NK cell- and γδ T cell-mediated graft-vs-leukemia effect has been evidenced with the success of allo-SCT with KIR/HLA mismatch in AML (13–17). Again, immune evasion occurs *via* various mechanisms [reviewed in Ref. (18)], including defects in the normal lymphopoiesis (19) and reduced expression of activating receptors, either through direct contact or *via* the secretion of immunosuppressive soluble factors such as TGFβ and indoleamine 2,3-dioxygenase (20) as well as shedding of soluble ligands (18). As for T cells, Tregs and MDSCs further impair NK cells effector functions (21, 22).

To date, among immune alterations described in AML patients at diagnosis, NK alterations are the most significant parameters correlated with prognosis (23, 24). Immune escape partly takes the form of NK cell subversion, which includes downregulation of NK triggering receptors such as NKp30, NKp46, DNAM-1, and NKG2D and upregulation of NK inhibitory receptors such as KIR and NKG2A (23–26). Therefore, and because NK cells are promising tools for therapeutic strategies, an exhaustive knowledge of NK cell dysfunctions in AML is mandatory. More recently, we and others [manuscript submitted; (27)] have evidenced a drastic reduction of immature NK cells in AML patients. However, a more comprehensive view of the NK cell maturation status of NK cells in AML is lacking.

Natural killer cell maturation is a multistep process marked by differential expression of several markers, among which CD56, CD16, NKG2A, KIR, and CD57 are of particular importance (28). First of all, CD56^bright^ NK cells expressing low levels of CD16 correspond to a transition between early immature CD56^bright^ CD16^−^ NK cells and CD56^dim^ CD16^+^ NK cells (29–32). Subsequently, NK cells lose expression of NKG2A and sequentially express KIR. Expression of CD57 marks the acquisition of high cytotoxic potential and decrease of proliferation capacities. Accordingly, NK cells display different functions during the maturation process, such as migration capacities, cytotoxic functions, cytokine/chemokine production, and response to cytokines (13, 24, 32). Given these functions are absolutely required for recognition and elimination of leukemic blasts, the clinical outcome may be affected by variations of sub-populations of NK cells with respect to maturation. For instance, increased NK maturation based on the percentage of CD57^+^ NK cells has been correlated with improved survival in both solid and hematologic malignancies (33). Such observation has also been validated in mouse models of lymphoma, AML, and melanoma, with interruption of functional maturation by tumors during NK-cell development (19, 27). Altogether, these findings confirm a general tendency of tumor cells to interfere with the development of cytotoxic anti-leukemia immune cells. In addition, CMV-induced NK maturation has been linked to the generation of CD56^dim^/CD57^+^/NKG2C^+^ NK cells defined as memory-like NK cells, and recent studies evidenced the anti-leukemic effect of this NK subpopulation (34–36).

We have recently reported that NK cells in AML patients display marked differences in NK maturation compared to healthy subjects, defining three distinct groups of patients according to NK maturation profiles (37). In this study, we extended the maturation profile of NK cells in AML to more mature NK cells such as memory-like NK cells in addition to the previously described stages of maturation in a large multicenter cohort allowing us to statistically examine the impact of maturation defects on the clinical outcome of patients.

## Patients and Methods

### Patients and Study Design

Baseline maturation profile on NK cells at diagnosis was assessed in a total of 87 patients from the LAM2006IR prospective multicenter randomized trial (NCT00860639) of the Groupe Ouest Est d’Etude des Leucémies Aiguës et autres Maladies du Sang (GOELAMS). Patient samples were collected between November 2007 and April 2012. All patients had previously untreated AML with intermediate-risk cytogenetics. Patients received conventional 3 + 7 induction chemotherapy with or without the addition of Gemtuzumab Ozogamicin (38). Patients with acute promyelocytic leukemia AML and patients above 66 years were excluded. The CMV status was not available. All participants gave written informed consent in accordance with the Declaration of Helsinki. The entire research procedure was approved by the ethical review boards from the IPC and the GOELAMS.

### Clinical Samples

Peripheral blood mononuclear cells (PBMCs) cryopreserved in 90%FCS/10%DMSO were obtained from randomly selected patients before induction chemotherapy and from healthy volunteers (HVs) (*N* = 19). Handling, conditioning, and storing of samples were performed by the FILOtheque AML (No. BB-0033-00073), tumor bank of the FILO group, Cochin Hospital, Paris.

### Flow Cytometry

A FACS LSR-Fortessa (BD Biosciences, San Jose, CA, USA) was used for flow cytometry. NK cells were immunostained with Krome Orange-conjugated anti-CD45, (ECD)-conjugated anti-CD3, allophycocyanin-alexafluor 700 (APC AF700)-conjugated anti-CD56, Phycoerythrin cyanin 7 (PC7)-conjugated anti-CD158b1,b2j, PC7-conjugated anti-CD158a,h (further referred to as KIR), Pacific Blue-conjugated anti-CD57, Phycoerythrin (PE)-conjugated NKp30, and Live/dead^®^ Near-IR (Thermo Fisher Scientific, Waltham, MA, USA). All the antibodies used in the study were a kind gift of Beckman-Coulter, Marseille, France.

### Mass Cytometry Analysis

Peripheral blood mononuclear cells were thawed and washed with RPMI with 10% FCS and incubated RPMI 2%FCS 1/10,000 Pierce^®^ Universal Nuclease 5 kU (Thermo Fisher Scientific, Waltham, MA, USA) at 37°C with 5% CO_2_ for 30 min. Cells were washed and stained with Cisplatin 0.1 M for dead cells exclusion. Cells were blocked with 0.5 mg/mL Human Fc Block (BD Bioscience). Two million PBMCs were stained 45 min at 4°C with the extracellular antibodies (Table S1 in Supplementary Material). Cells were washed and barcoded with the Cell-ID™ 20-Plex Pd *Barcoding Kit* (Fluidigm) according to the manufacturer’s recommendations. Cells were washed, and samples were combined and stained with metal-labeled anti-PE secondary antibodies 30 min at 4°. Cells were washed and permeabilized with Foxp3 Staining Buffer Set (eBioscience, San Diego, CA, USA) 40 min at 4°C. Cells were incubated with 0.5 mg/mL Human Fc Block 40 min at 4°C, and stained 40 min at 4°C in Foxp3 Staining Buffer with the intracellular antibodies (Table S1 in Supplementary Material). Then, cells were washed and labeled overnight with 125 nM iridium intercalator (Fluidigm) in Cytofix (BD Biosciences). Finally, cells were diluted in EQTM Four Element Calibration Beads (Fluidigm) before acquisition on a CyTOF2^®^ instrument (Fluidigm).

### Patient Classification

Patients were classified according to maturation markers expression as previously described (37). Briefly, patients and the mean of HV were clustered according to the percentages of NK cells represented in the CD56^bright^, KIR^−^/CD57^−^, KIR^+^/CD57^−^, KIR^−^/CD57^+^, and KIR^+^/CD57^+^ clusters with MeV software using unsupervised hierarchical clustering (HClust, Euclidian distance).

### Statistical Analyses

Statistical analyses were carried out using Graph Pad Prism (Graph Pad Software, San Diego, CA, USA) and SPSS (SPSS Software, Chicago, IL, USA). For multiple comparisons, a Kruskal–Wallis test was used followed by a Dunn’s post-test. Association between variables was assessed using the Spearman correlation coefficient. For survival analyses, overall survival (OS) was defined as the time from diagnosis until death from any cause, and relapse-free survival (RFS) as the time between induction and relapse or death, whatever occurred first. Patients without an event were censored at the time of their last follow-up. Survival times were estimated by Kaplan–Meier method and compared using the log-rank test. A multivariate Cox regression model was used to assess the predictive value of NKp30 expression while adjusting for other prognostic factors [age at diagnosis, European Leukemia Net (ELN), leukocytosis, and allo-SCT as a time-dependent covariate]. The limit of significance was set at *P* < 0.05.

## Results

### Baseline Patient Characteristics

The patient characteristics, stratified by NK maturation profile, are summarized in Table 1. All patients had intermediate-risk cytogenetics. The mean age (±SD) at induction was 46.9 years (±11.3). Median follow-up after diagnosis was 24.9 months. Cytogenetic classification and ELN genetic classification (39) (FLT3/CEBPα/NPM1 mutational status) were routinely determined in the Biopathology departments of the centers involved in this study.

**Table 1:** **Baseline patients characteristics (1/2)**.

Characteristic		All	Hyper-maturation	Intermediate maturation	Hypo-maturation
Patients, no.	*N*	87	21 (24.1)	58 (66.7)	8 (9.2)
Age at diagnosis	Mean (SD)	46.9 (11.3)	49.8 (9.9)	46.0 (11.6)	45.3 (12.3)
Sex ratio (M/F)		1.2	2.5	0.9	1.7
FAB category	*N* (%)				
M0		1 (1.1)	0 (0.0)	1 (1.7)	0 (0.0)
M1		13 (14.9)	3 (14.3)	9 (15.5)	1 (12.5)
M1/M2		2 (2.3)	1 (4.8)	1 (1.7)	0 (0.0)
M2		19 (21.8)	3 (14.3)	14 (24.1)	2 (25.0)
M3		0 (0.0)	0 (0.0)	0 (0.0)	0 (0.0)
M4		17 (19.5)	2 (9.5)	13 (22.4)	2 (25.0)
M4/M5		1 (1.1)	0 (0.0)	1 (1.7)	0 (0.0)
M5		22 (25.3)	8 (38.1)	11 (19.0)	3 (37.5)
M6		1 (1.1)	0 (0.0)	1 (1.7)	0 (0.0)
M7		0 (0.0)	0 (0.0)	0 (0.0)	0 (0.0)
Unclassified		1 (1.1)	0 (0.0)	1 (1.7)	0 (0.0)
NA		10 (11.5)	4 (19.0)	6 (10.3)	0 (0.0)
Status at diagnosis	*N* (%)				
*De novo*		86 (89.9)	20 (95.2)	58 (100.0)	8 (100.0)
t-AML		1 (1.1)	1 (4.8)	0 (0.0)	0 (0.0)
White blood cell (10^9^ cells/L)	Median (SD)	24.7 (28.5)	19.7 (37.7)	29.2 (22.9)	16.8 (41.5)
Cytogenetic prognosis	*N* (%)				
Favorable		0 (0.0)	0 (0.0)	0 (0.0)	0 (0.0)
Intermediate		87 (100.0)	21 (100.0)	58 (100.0)	8 (100.0)
Adverse		0 (0.0)	0 (0.0)	0 (0.0)	0 (0.0)
Mutations in intermediate group	*N* (%)				
FLT3 ITD^mut^		32 (36.8)	7 (33.3)	20 (34.5)	5 (62.5)
CEBPα^mut^/FLT3^wt^		26 (29.9)	6 (28.6)	20 (34.5)	0 (0.0)
NPM1^mut^/FLT3^wt^		5 (5.7)	0 (0.0)	5 (8.6)	0 (0.0)
ELN	*N* (%)				
Favorable		30 (26.4)	6 (28.6)	24 (41.4)	0 (0.0)
Intermediate		57 (65.5)	15 (71.4)	34 (58.6)	8 (100)
Adverse		0 (0.0)	0 (0.0)	0 (0.0)	0 (0.0)
Blasts (blood) at diagnosis	Mean (SD)	44.3 (33.1)	50.5 (40.4)	41.8 (30.5)	46.7 (33.1)
Blasts (BM) at diagnosis	Mean (SD)	62.2 (23.3)	65.5 (23.9)	59.0 (23.5)	75.8 (14.8)
Response at day 15	*N* (%)	48 (55.2)	11 (52.4)	35 (60.3)	2 (25.0)
No response at day 15		32 (36.8)	7 (33.3)	19 (32.8)	6 (75.0)
NA (not evaluable or induction death)		7 (8.0)	3 (14.3)	4 (6.9)	0 (0.0)
Post-induction CR	*N* (%)	73 (83.9)	17 (81.0)	50 (86.2)	6 (75.0)
No post-induction CR		14 (16.1)	4 (19.0)	8 (13.7)	2 (25.0)
Induction death		5 (5.7)	2 (9.5)	2 (3.4)	1 (12.5)
No CR achieved		9 (10.3)	2 (9.5)	6 (10.3)	1 (12.5)
Nb of induction for CR	*N* (%)				
1		53 (60.9)	12 (71.4)	36 (62.1)	2 (25.0)
2		15 (17.2)	2 (9.5)	10 (17.2)	3 (37.5)
3		4 (4.6)	0 (0.0)	3 (5.2)	1 (12.5)
Allo-SCT	*N* (%)	26 (29.9)	6 (28.6)	15 (25.9)	5 (62.5)

*Allo-SCT, allogeneic stem cell transplantation; BM, bone marrow; CR, complete remission; FAB, French–American–British classification; M, male; F, female; ITD, internal tandem duplication; NA, not available; Nb, number; t-AML, therapy-related AML*.

### AML Patients Present Distinct Maturation Profiles

**Figure 1:**
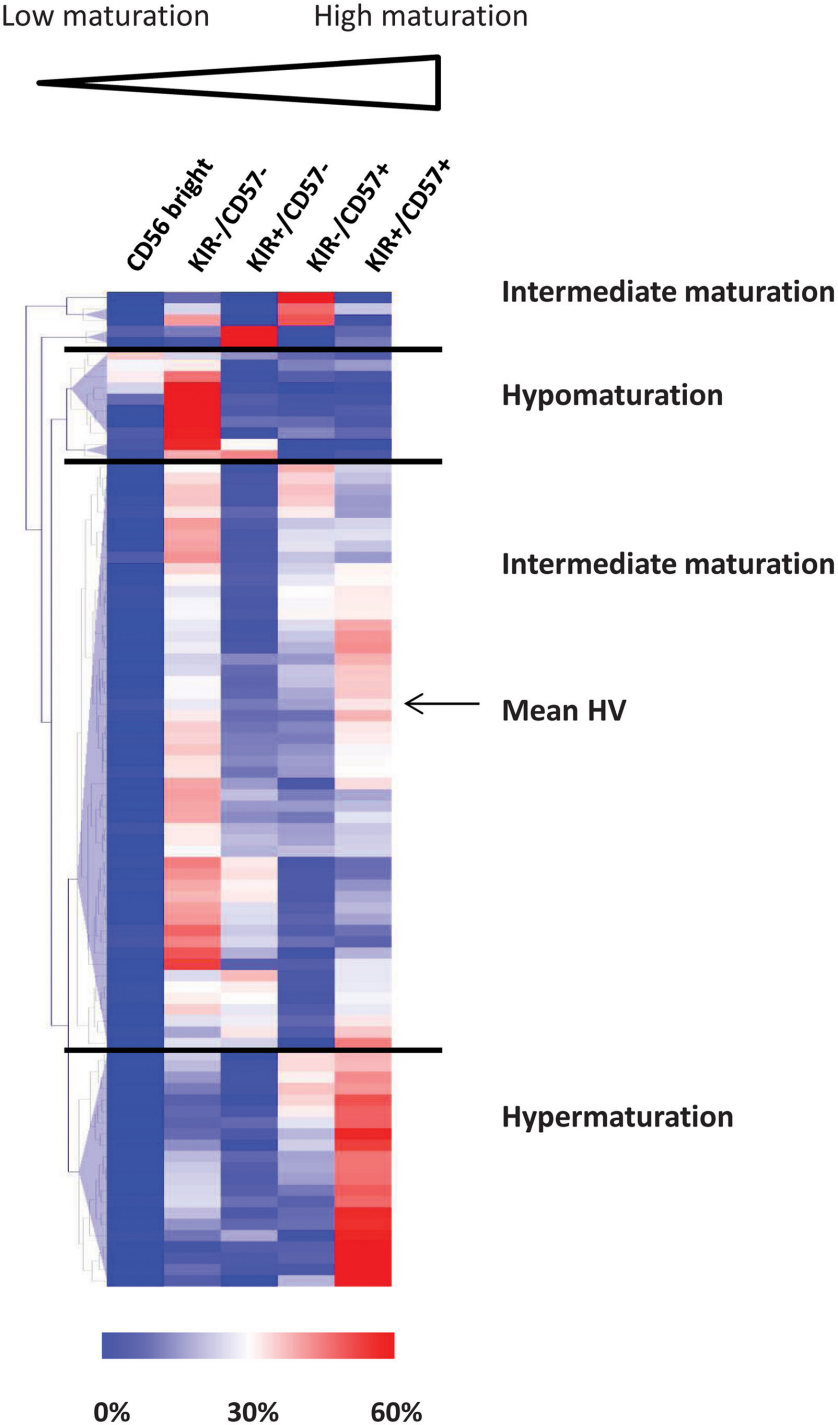
**Unsupervised classification of acute myeloid leukemia patients according to natural killer (NK) maturation profiles**. Patients and HVs were classified according to the percentage of NK cells represented in the CD56^bright^, KIR^−^/CD57^−^, KIR^+^/CD57^−^, KIR^−^/CD57^+^, and KIR^+^/CD57^+^ clusters using hierarchical clustering (Euclidian distance). HVs, healthy volunteers.

In humans, four parameters define NK cell subsets according to the expression of NKG2A, KIR, CD57, and CD56 (28, 40, 41). CD56^bright^ phenotype defines the most immature subset of circulating NK cells. In CD56^dim^ NK cells, loss of NKG2A and acquisition of KIR and CD57 define several maturation stages. Baseline expression of the maturation parameters CD56, KIR, and CD57 on NK cells was assessed by flow cytometry. As previously described, NKG2A was not informative in NK cell cluster definition and was therefore omitted in the clustering process (37). Using unsupervised HClust, patients and HVs were classified according to the percentages of NK cells represented in the CD56^bright^, CD56^dim^/KIR^−^/CD57^−^, CD56^dim^/KIR^+^/CD57^−^, CD56^dim^/KIR^−^/CD57^+^, and CD56^dim^/KIR^+^/CD57^+^ clusters (Figure 1). This representation enables to define three distinct groups of patients. Among the 87 patients, 8 (9.2%) had low NK maturation profile, with most NK cells in the CD56^bright^ and CD56^dim^/KIR^−^/CD57^−^ immature clusters (hypomaturation group); 21 patients (24.1%) had hyper maturation profile, with most NK cells in the CD56^dim^/KIR^+^/CD57^+^ cluster; 58 patients (66.7%) had intermediate maturation profile, with NK cells distributed into the CD56^dim^/KIR^−/+^/CD57^+/−^ clusters (Table 1).

In conclusion, we observed that NK cells in AML patients display marked differences with regards to maturation, defining three distinct groups of patients, thus confirming previous results obtained in our pilot explorative cohort (37).

### Maturation Profiles in Peripheral Blood Are Representative of NK Maturation in the Bone Marrow (BM)

**Figure 2:**
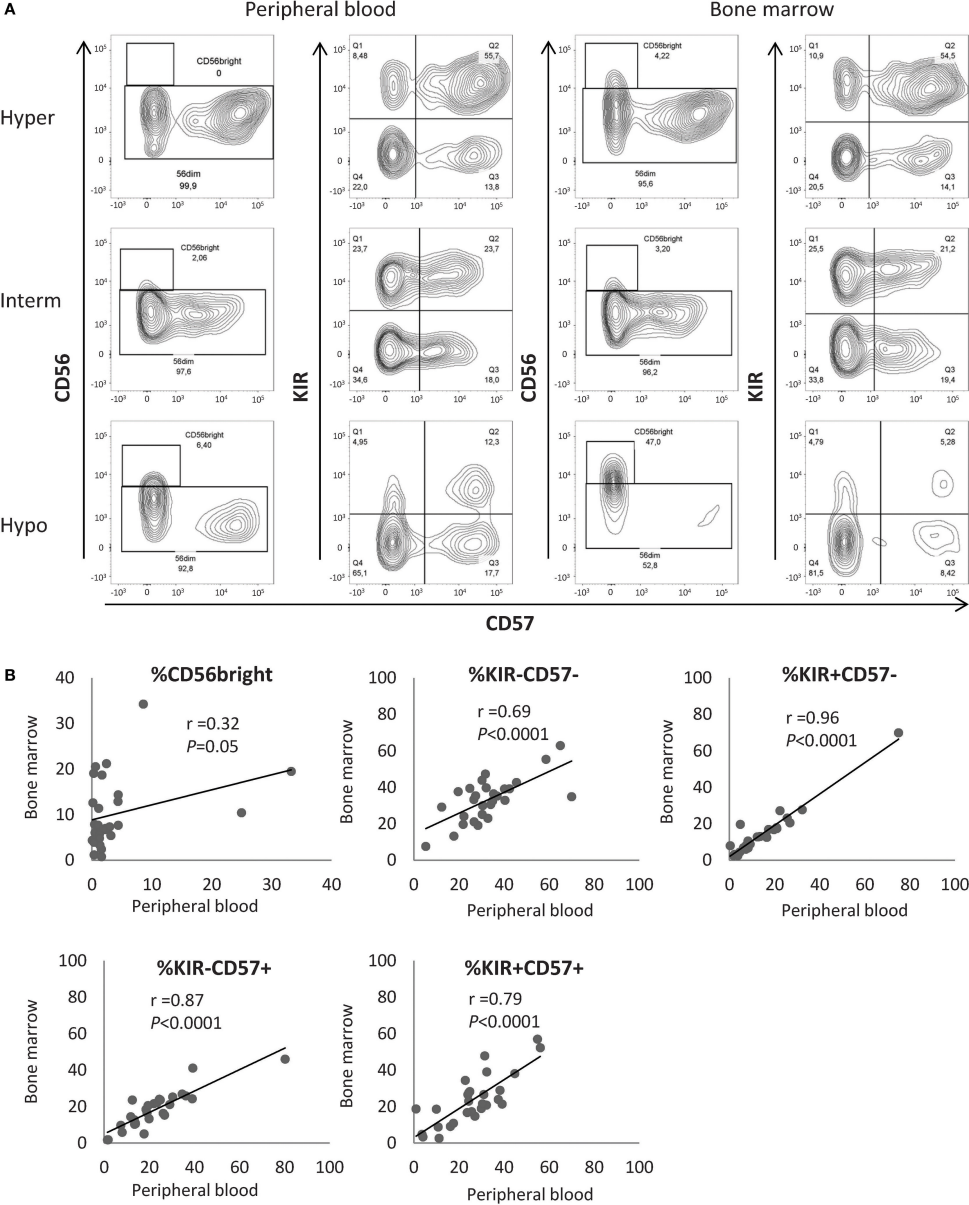
**Maturation profiles in the peripheral blood are representative of natural killer (NK) maturation profiles in the bone marrow (BM)**. **(A)** Examples of maturation profiles on appariated peripheral blood and BM samples by flow cytometry. **(B)** Correlation between the frequencies of NK cells in the different NK maturation clusters (Pearson correlation). Hyper, hypermaturation; Hypo, hypomaturation; Interm, intermediate maturation.

As AML develops in the BM before disseminating in the periphery, we hypothesized that NK cell defects in the BM must be at least as pronounced as compared to NK cells from PB. Therefore, we tested whether the measurement of NK maturation parameters in PB accurately reflects NK maturation in BM. For 28 patients of the cohort, cryopreserved BM samples were available. BM NK cells were analyzed by flow cytometry according to the protocol described for peripheral PBMCs. In BM, NK maturation profiles were similar to the maturation profiles in PB (Figure 2A). We compared the frequency of NK cells in the CD56^bright^, CD56^dim^/KIR^−^/CD57^−^, CD56^dim^/KIR^+^/CD57^−^, CD56^dim^/KIR^−^/CD57^+^, and CD56^dim^/KIR^+^/CD57^+^ clusters in the PB and in the BM with the Pearson correlation coefficient. As expected, BM contained more CD56^bright^ NK cells compared to PB (10.1 vs 3.9%, respectively; *P* < 0.001) and a moderate correlation was observed between the frequencies of CD56^bright^ NK cells in PB and in BM (*r*^2^ = 0.32, *P* = 0.05, Figure 2B). For all the other clusters of NK cells, correlations coefficients were high (CD56^dim^/KIR^−^/CD57^−^: *r*^2^ = 0.69, *P* < 0.0001; CD56^dim^/KIR^+^/CD57^−^: *r*^2^ = 0.96, *P* < 0.0001; CD56^dim^/KIR^−^/CD57^+^: *r*^2^ = 0.87, *P* < 0.0001; CD56^dim^/KIR^+^/CD57^+^: *r*^2^ = 0.79, *P* < 0.0001), with no significant difference between PB and BM (Figure 2B). Thus, the maturation profile of NK cells in PB is representative of the maturation status of NK cells in BM.

### Defective NK Maturation Impacts Clinical Outcome

**Figure 3:**
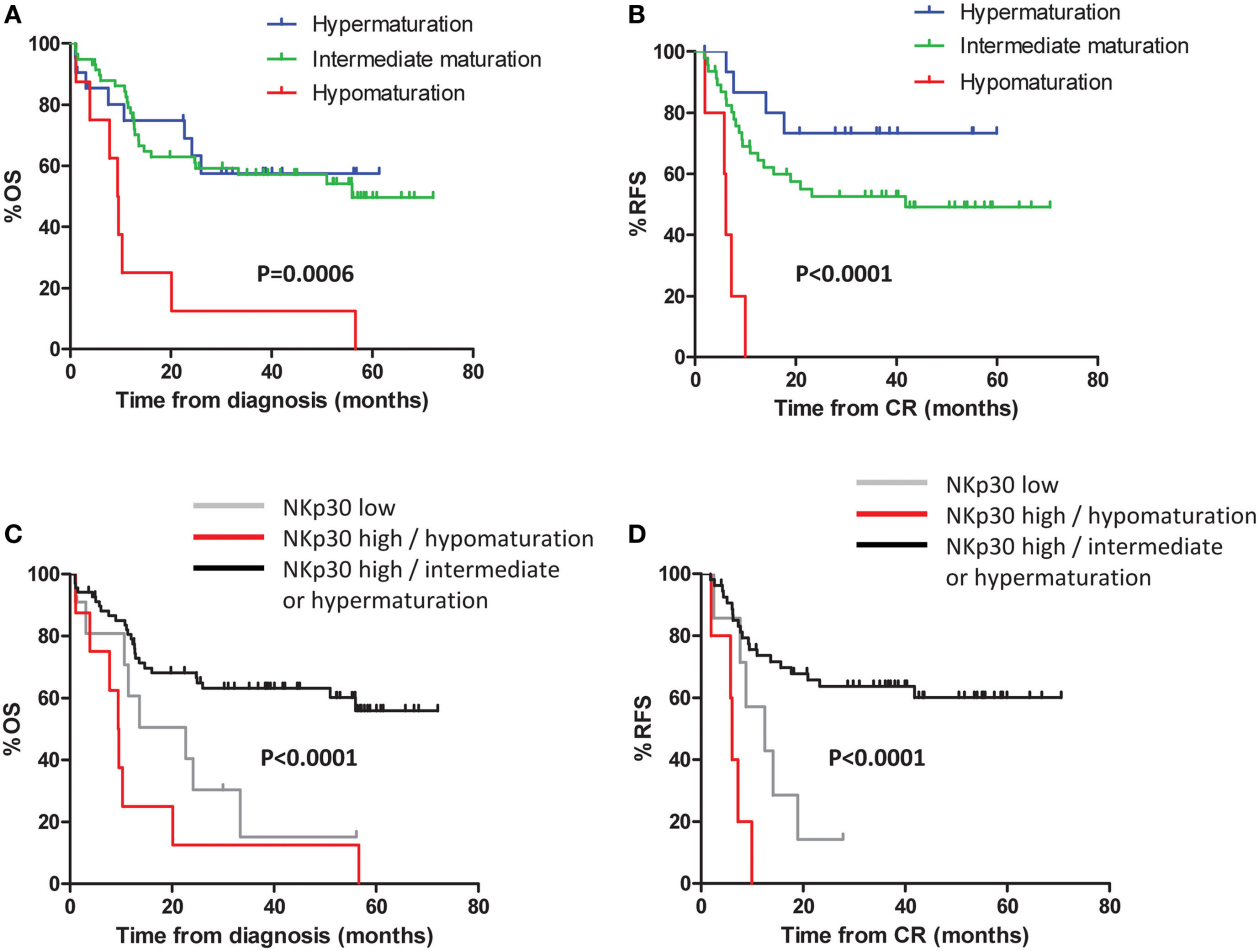
**Defective natural killer (NK) maturation impacts clinical outcome**. Kaplan–Meier curves of overall survival (OS) **(A)** and event-free survival **(B)** by NK maturation profile at diagnosis. Kaplan–Meier curves **(C,D)** display OS and relapse-free survival (RFS) by NKp30 status refined by NK maturation status: patients were classified in two groups according to NKp30 expression. Among patients with high NKp30 expression, patients were divided into two groups according to NK maturation.

Patients were divided into three groups as defined above (NK hypomaturation, intermediate maturation, and hypermaturation). In univariate analysis, significant differences in OS (*P* = 0.0006) and RFS (*P* < 0.0001) were observed among the different groups (Figures 3A,B). Patients with hypomaturation profile had reduced OS, with 3-year OS rates of 12.5 vs 57.1 and 57.4% for patients with intermediate and hypermaturation, respectively. Consistently, patients with hypomaturation profile had reduced RFS, with 3-year RFS rates of 0 vs 52.6 and 73.3% for patients with intermediate and hypermaturation, respectively. In multivariate Cox regression models, NK hypomaturation remained significantly associated with reduced OS and RFS, independent of other factors [hazard ratio (HR) = 4.15, *P* = 0.004 and HR = 8.23, *P* = 0.003, respectively] (Table 2).

**Table 2:** **Cox regression**.

	Multivariate HR for OS			Multivariate HR for RFS		
Variable	HR	95% CI	*P*	HR	95% CI	*P*
**Age at diagnosis**						
<50	Reference			Reference		
≥50	1.03	0.54–1.96	0.93	0.79	0.38–1.62	0.51
**ELN**						
Favorable	Reference			Reference		
Intermediate	2.37	1.08–5.19	0.03	1.58	0.69–3.62	0.28
**Leucocytosis at diagnosis**						
<50 G/L	Reference			Reference		
≥50 G/L	1.01	0.49–2.06	0.99	0.93	0.41–2.11	0.86
**Consolidation**						
No allo-SCT	Reference			Reference		
Allo-SCT	0.32	0.13–0.79	0.01	0.50	0.19–1.33	0.17
**Natural killer (NK) maturation profile**						
Hyper/intermediate	Reference			Reference		
Hypomaturation	4.15	1.58–10.87	0.004	8.23	2.51–26.94	<0.001

*Multivariate Cox regression models were used to assess the predictive value of NK maturation profile while adjusting for the prognostic factors in the population (age at diagnosis, ELN, leukocytosis, and allo-SCT as a time-dependent covariate)*.

*Allo-SCT, allogeneic stem cell transplantation; CI, confidence interval; RFS, relapse-free survival; ELN, European Leukemia Net genetic classification; HR, hazard ratio; OS, overall survival*.

Interestingly, the group of patients with NK hypomaturation did not correspond to the group of patients with NKp30^low^ phenotype, a NK triggering receptor downregulated in AML. Since NKp30^low^ phenotype is associated with adverse clinical outcome in AML [(23); submitted manuscript], we combined maturation-based classification with NKp30 status (data not shown). The following groups were assembled: NKp30^low^ patients irrespective of maturation profile, NKp30^high^/hypomaturation profile patients, and NKp30^high^/intermediate or hypermaturation profile patients (Figures 3C,D). The NKp30^low^ and NKp30^high^/hypomaturation groups of patients displayed the worst clinical outcome with lower OS (*P* < 0.0001) and RFS (*P* < 0.0001).

### NK Hypomaturation Profile Is Associated with a Lack of Generation of Memory-Like NK Cells in AML

**Figure 4:**
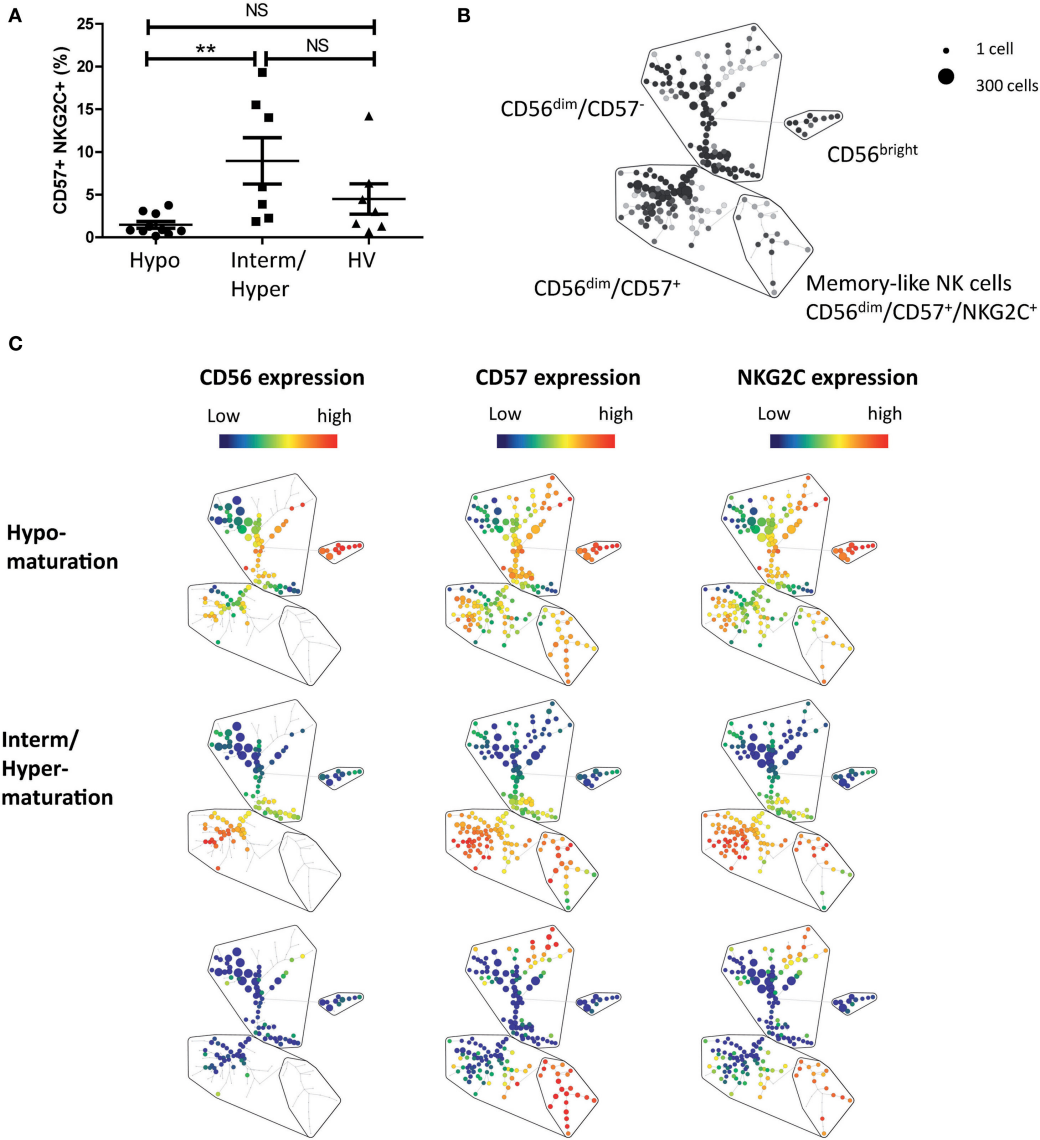
**Natural killer (NK) hypomaturation profile is associated low frequency of memory-like NK cells**. NK alterations associated with the different maturation phenotypes were explored by mass cytometry. Peripheral blood mononuclear cells from 17 additional patients with newly diagnosed acute myeloid leukemia and 7 healthy volunteers (HVs) were analyzed by mass cytometry. Memory-like NK cells were defined as CD56^dim^/CD57^+^/NKG2C^+^ NK cells. **(A)** Analysis of the percentage of memory-like NK cells by NK maturation group. **(B)** Gating strategy of memory-like NK cells on a Spade tree. **(C)** Representative Spade trees of patients and HVs. The first column represents CD56 expression, which defines the cluster of CD56^bright^ NK cells. The second column displays CD57 expression, which divides CD56^dim^ NK cells into CD57^+^ and CD57^−^ NK cells. The third column displays NKG2C expression, which enables to identify the cluster of memory-like NK cells, defined as CD56^dim^/CD57^+^/NKG2C^+^ NK cells.

Maturation of NK cells is not terminated with the expression of CD57. Hence, recent studies have identified a subset of NK cells expressing CD57 and NKG2C receptors and associated with memory-like properties (42). We next appended the maturation status of patients with the analysis of CD56^dim^/CD57^+^/NKG2C^+^ NK cells. PBMCs from 17 additional patients with newly diagnosed AML and 7 HVs were analyzed by mass cytometry (Figure 4). Classification of patients according to NK maturation profiles was performed as described above and allowed retrieving subgroups of patients based on maturation profile. We then assessed, in the CD56^dim^ clusters, the percentage of memory-like NK cells, defined as CD56^dim^/CD57^+^/NKG2C^+^ NK cells (34, 43). The frequency of memory-like NK cells was higher, but non-significant, in patients with intermediate or hypermaturation than in healthy subjects (9.0 vs 4.5%, respectively). However, there was a huge difference in frequency of memory-like NK cells between patients with NK hypomaturation profile compared to patients with intermediate or hypermaturation profile (1.5 vs 9.0%, respectively, *P* < 0.01) (Figures 4A,B).

## Discussion

Understanding immune alterations in cancer patients is a major challenge, with exponential applications in terms of therapeutic targeting. However, improved knowledge of interactions between immunity and malignant cells is critical to improve immunotherapeutic strategies.

Natural killer cells are potent innate immune cells that were first described for their ability to kill cancer cells without prior sensitization (30). Among parameters involved in NK functionality, maturation is an important process, during which NK cells acquire higher cytotoxic potential (44). In the case of patients with AML, we have recently reported the existence of distinct NK maturation profiles (37). In this study, we defined three groups of patients based on unsupervised analysis of CD56, KIR, and CD57 expression, with either NK maturation blockade at early stages, hypermaturation, or intermediate maturation profiles. However, the potential impact on clinical outcome of such observations remained to be tested on a larger cohort of patients.

In this study, we took advantage of frozen samples from the FILOtheque (French Innovative Leukemia Organization, Paris, France) during the course of a clinical trial of AML of intermediate prognosis. These samples were fully annotated. We confirm in a large multicenter cohort the existence of three groups of patients with distinct NK maturation profiles. NK maturation profiles are not or slightly modified after complete remission (CR) (Figure S1 in Supplementary Material), by contrast to other tumor-induced NK alterations such as NK triggering receptor expression, whose expression is normalized at CR [(23); Rey et al., in revision]. In survival analyses, the group of patients with enriched NK cells at early stage of maturation had an adverse clinical outcome compared to the other groups, with a median OS 9.5 vs 56.0 months for patients with intermediate NK maturation (undefined for patients with NK hypermaturation). In addition, we showed that NK maturation blockade is an independent predictor of adverse clinical outcome in multivariate analysis, even when accounting for the potential effects of allo-SCT, and despite heterogeneous distribution of NPM1 mutations in the different groups.

We then compared the prognostic value of NK maturation with the one of NKp30 status. HRs were lower for NK maturation status, suggesting that this parameter might be more informative for outcome prediction (Figures S2A–D in Supplementary Material). However, both classifications identify patients with dramatic clinical outcome. Importantly, no patient with NK maturation blockade had low NKp30 expression (Figure S2H in Supplementary Material), which indicates that these classifications are not redundant. Thus, by combining both parameters, we improved the discrimination of patients with adverse clinical outcome; in this system, NK maturation status enables identification of patients with adverse prognosis that was not identified with the classification based on NKp30 alone. Such classification would be extremely relevant, in particular in the group of patients with intermediate-risk cytogenetic and intermediate-risk ELN, as in this study, for which definition of prognostic factors remains elusive (45). However, the prognostic significance of this classification is explorative and has to be confirmed on an independent cohort of patients. In addition, functional assays should be performed on NK cells from each group of patients to assess the cytotoxic capacity and the production of CD107α, TNFα, and IFNγ of NK cells according to the maturation status. This step would provide final evidence of the link between NK maturation blockade and adverse clinical outcome.

On the other hand, NK maturation profile is partially based on the frequency of CD57 on NK cells. Since high CD57 expression on NK cells has been correlated with favorable prognosis in various cancers [reviewed in Ref. (33)], the classification based on NK maturation might be strongly influenced, if not completely carried out, by CD57 expression. When comparing the classifications based on NK maturation status vs frequencies of CD57^+^ NK cells, we evidence that, although significant, the classification based on CD57 alone is less performant compared to NK maturation profiles, with lower HR and lower significance (Figures S2E,F in Supplementary Material). Moreover, the classification based on CD56/CD57/KIR expression is unsupervised, which has the strong advantage to avoid the step of threshold determination, which might lead to overfitting of prediction models based on a single marker.

Beyond the increase of cytotoxic properties, NK maturation has been linked to the generation of memory NK cells (40, 46). Recent studies demonstrated anti-leukemic effect of memory-like NK cells, with enhanced control of AML in mice and humans (35, 36). Consistently, in our study, patients with NK maturation blockade had lower frequencies of memory-like NK cells (1.5 vs 9.0% for patients with hyper or intermediate NK maturation), defined as CD56^dim^/CD57^+^/NKG2C^+^ NK cells (43). We can speculate that the adverse clinical outcome in patients with low frequency of mature NK cells might be related to the lack of NK memory response. However, this hypothesis is based on an observation on a limited number of patients and warrants confirmation on a larger cohort. Importantly, NK maturation as well as the frequency of memory NK cells must be analyzed with regards to CMV status, which has been correlated with increased NK maturation and expansion of memory NK cells (34).

Besides alterations on NK cells, T cells alterations in AML have been described (8), some of which having been associated with inferior clinical outcome (9). Thus, there is a need for overall picture of innate and adaptive immune landscape in AML; technical advances in flow and mass cytometry now enable to dissect the complexity of the immune system and will soon provide the bases for fully integrated analysis of immune alterations in AML (47).

In conclusion, besides classical alterations of NK triggering and inhibitory receptors expression in AML, we confirm that the homeostasis of NK maturation can be modified in the context of AML, notably with a deep maturation blockade in a fraction of patients for which clinical outcome is dramatic. Furthermore, low NK maturation profile is associated with the absence of generation of memory-like NK cells, which may imply reduced anti-opportunistic disease responses. In addition, this phenotype reveals potential information for risk stratification based on expression of the triggering receptor NKp30. Therefore, NK maturation status is likely to be informative in prognostic immune signatures based on NK parameters, with potential applications in terms of patient stratification. The elucidation of mechanisms involved in the emergence of this particular NK cell phenotype will provide new opportunities to enhance NK cell functions in AML patients.

## Ethics Statement

Name of the ethics committee that approved the study: all participants gave written informed consent in accordance with the Declaration of Helsinki. The entire research procedure was approved by the ethical review boards from the IPC and the GOELAMS. Paoli Calmettes Institute Ethic Committee. Groupe Ouest Est d’Etude des Leucémies Aiguës et autres Maladies du Sang (GOELAMS) Ethic Committee. FILO group (French Innovative Leukemia Organization, Paris, France). No. BB-0033-00073. LAM2006IR prospective multicenter randomized trial (NCT00860639).

## Author Contributions

All authors: conception and design of the work; acquisition, analysis, and interpretation of data; drafting and revising of the manuscript; final approval of the manuscript and agreement to be accountable for all aspects of the work in ensuring that questions related to the accuracy or integrity of any part of the work are appropriately investigated and resolved.

## Conflict of Interest Statement

A-SC, CF, and CA: Patents, Royalties, Other Intellectual Property: INSERM Transfert. FO, J-FH-B, SG, CL, PC-L, JD, AT, HL, MM, and EG: no relationship to disclose. JR: Consulting or Advisory Role: Novartis. Travel, Accommodations, Expenses: Novartis. GB and EG: Employment: Beckman Coulter Immunotech. The role of this author was to contribute to the design of the flow cytometry analysis, in particular to the reagent panels design. NI: disclosure has been completed by ASCO’s COI system. NV: Consulting or Advisory Role: Novartis, Roche. Travel, Accommodations, Expenses: Amgen, Novartis. Patents, Royalties, Other Intellectual Property: INSERM Transfert. DO: Stock or Other Ownership: Imcheck Therapeutics. Research Funding: GSK. Patents, Royalties, Other Intellectual Property: INSERM Transfert, GSK. JN: Consulting or Advisory Role: Abreos Biosciences.
