# Supplementary material for: Natural Killer Defective Maturation Is Associated with Adverse Clinical Outcome in Patients with Acute Myeloid Leukemia
Source: Front Immunol. 2017 May 29;8:573. doi: 10.3389/fimmu.2017.00573 (PMC5447002; doi:10.3389/fimmu.2017.00573)
Supplement: Supplementary file 1 [file Table_1.docx]

Supplementary Table 1 - Mass Cytometry panel

| Antigen | Metal/fluorochrome |  | Company |
| --- | --- | --- | --- |
| CD45 | 89Y | Extracellular | Fluidigm |
| CD3 | 115In | Extracellular | CRCM* |
| CD19 | 142Nd | Extracellular | Fluidigm |
| CD45RA | 143Nd | Extracellular | Fluidigm |
| CD158b1/b2j | 154Sm | Extracellular | Beckman Coulter* |
| NKG2A | 165Ho | Extracellular | Beckman Coulter* |
| CD158a/h | 168Er | Extracellular | Beckman Coulter* |
| NKp30 | 169Tm | Extracellular | Beckman Coulter* |
| NKG2C | 170Er | Extracellular | Miltenyi* |
| CD57 | 172Yb | Extracellular | Fluidigm |
| CD34 | 174Yb | Extracellular | Biolegend* |
| CD56 | 176Yb | Extracellular | Fluidigm |
| CD13 | PE | Extracellular | BD Biosciences* |
| CD33 | PE | Extracellular | Beckman Coulter* |
| Anti-PE | 156Gd | Extracellular | Fluidigm |

* Purified antibodies conjugated with [Maxpar® Antibody Labeling Kit](https://www.fluidigm.com/binaries/content/documents/fluidigm/resources/maxpar-antibody-labeling-kit/maxpar-antibody-labeling-kit/fluidigm%3Afile) (Fluidigm)

Abbreviations: PE: phycoerythrin
