# Supplementary figures and images for: Natural Killer Defective Maturation Is Associated with Adverse Clinical Outcome in Patients with Acute Myeloid Leukemia
Source: Front Immunol. 2017 May 29;8:573. doi: 10.3389/fimmu.2017.00573 (PMC5447002; doi:10.3389/fimmu.2017.00573)

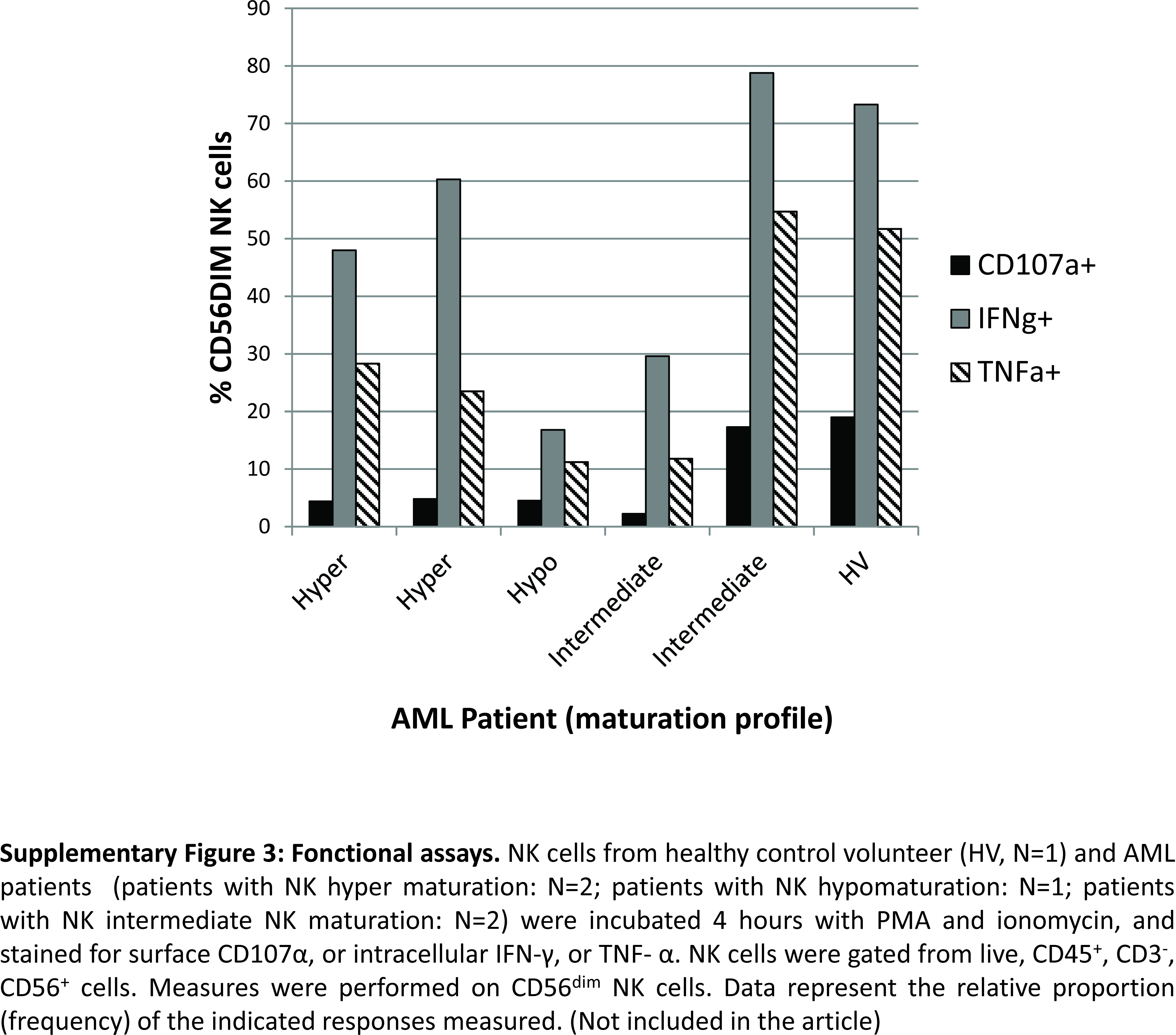

Supplement: Supplementary file 4 [file Image_3.jpeg]
